# A Rare Case of Cutaneous Leishmaniasis Presenting to the Emergency Department of a Large Community Hospital

**DOI:** 10.7759/cureus.56658

**Published:** 2024-03-21

**Authors:** Cally Wollet, Melody L Milliron

**Affiliations:** 1 Emergency Medicine, Allegheny Health Network, Saint Vincent Hospital, Erie, USA

**Keywords:** cutaneous leishmaniasis, multiple cutaneous ulcers, amphotericin b, leishmaniasis, parasitic infection, travel medicine

## Abstract

Cutaneous leishmaniasis should be considered a possible cause of skin ulcers in a patient who has traveled abroad recently and comes to the emergency department (ED) for an assessment. Before getting an accurate diagnosis, ED assessment, and proper treatment with intravenous amphotericin B, the patient presented to several other healthcare providers. This case displays the importance of a multidisciplinary approach with consultation from infectious diseases to determine an accurate diagnosis and effective treatment plan for patients with cutaneous leishmaniasis.

## Introduction

Leishmaniasis is a parasitic infection usually transmitted through the bites of female phlebotomine sandflies. There are three forms of leishmaniasis: visceral (VL), cutaneous (CL), and mucocutaneous (MCL). The most common type of leishmaniasis in the Americas is CL, caused by the species *Leishmania braziliensis* and *Leishmania mexicana* [1,2]. Disseminated cutaneous leishmaniasis (DCL) is a form of cutaneous leishmaniasis characterized by multiple lesions in various body regions.

The Centers for Disease Control and Prevention (CDC) estimates the number of new cases of CL per year is as many as 1.2 million, with most cases in the United States seen in patients who travel or immigrate [3]. Some of the symptoms of DCL include non-ulcerative cutaneous skin lesions that may grow diffusely. These lesions are most likely due to the overproduction of B cells and inadequate cytotoxic T cell response [4]. Prevention methods include insect repellent, minimizing the amount of exposed skin when outside, and insecticide sprays indoors [3].

Diagnosis of CL is made by skin biopsy. The sample will display macrophages, amastigotes, and rod-shaped kinetoplast. The kinetoplast is the most important feature for confident diagnosis of leishmaniasis [3]. The mainstay of treatment for DCL includes amphotericin B, either amphotericin B lipid complex (ABLC), liposomal amphotericin B (L-AmB), or amphotericin B colloidal dispersion (ABCD), with no significant difference in efficacy between the treatments [5]. Unlike other infectious diseases, earlier treatment does not necessarily equal better outcomes. In most cases, there is a better prognosis when the patient starts treatment after cutaneous lesions have already appeared [6]. The lesions show a clear indication of the presence of the disease, allowing for more accurate diagnosis and targeted treatment.

## Case presentation

A 35-year-old male presented to the ED with a fever and wounds on his hands. He had previously been evaluated by other specialists, including infectious disease. The patient stated that he was in Honduras three months prior during the summer, when he reported being bitten by several flying insects. Soon after this insect exposure in Honduras, he developed very tender ulcers on his hands, arms, left hip, back, and posterior scalp. Initially, he was evaluated in Honduras and received two grams of intramuscular ceftriaxone. However, the painful ulcers did not resolve, so when he arrived in the United States, he presented to urgent care. On this evaluation, he was given trimethoprim-sulfamethoxazole (TMP-SMX) and topical mupirocin without any improvement. Eventually, he was seen by an infectious disease specialist, who referred him for a biopsy of the ulcers. Following the biopsy, the patient had worsening symptoms, including hand swelling and fever. Therefore, the infectious disease physician referred the patient to the ED due to concerns about DCL.

**Figure 1:**
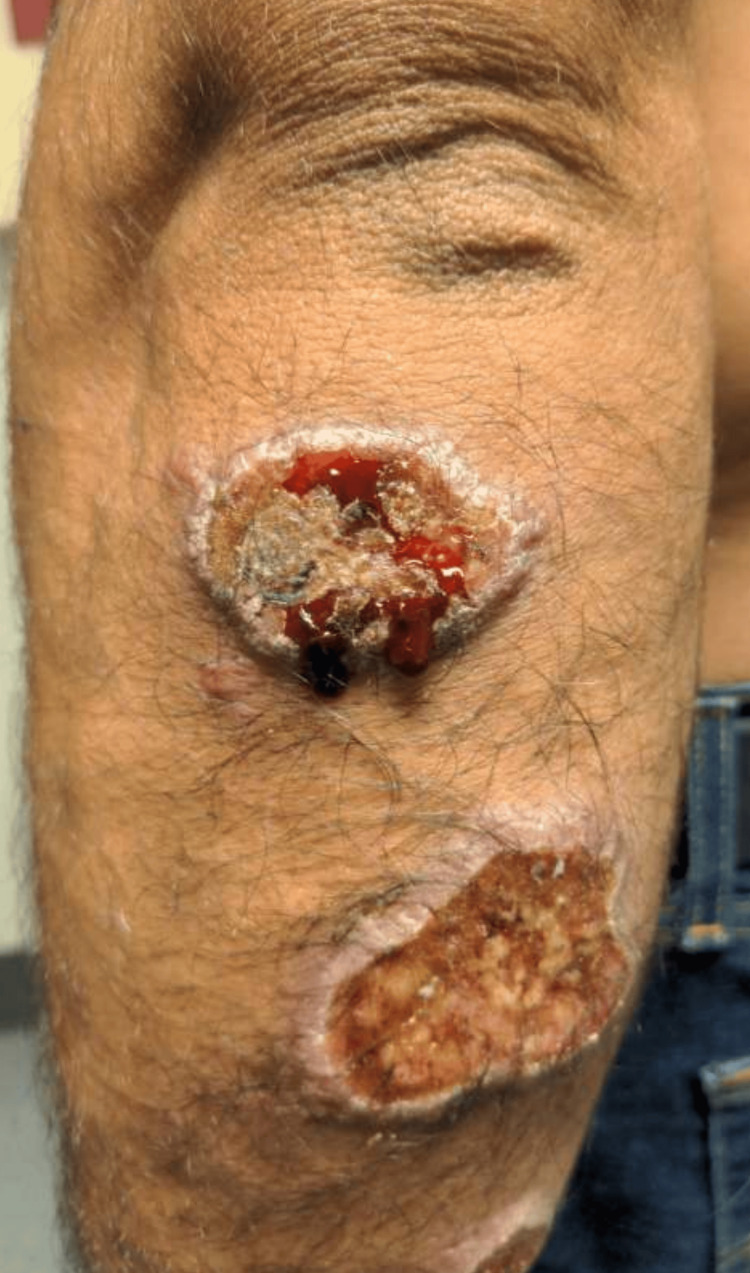
Photograph of the left elbow lesions.

A physical exam showed multiple superficial ulcerations on the patient’s hands, arms, left hip, back, right posterior scalp, and left elbow (Figure 1). After lab investigation (Table 1), infectious disease was consulted. They recommended amphotericin B 3 mg/kg intravenously in the ED, and admission for seven days for continued parenteral amphotericin. The local health department and the CDC were informed of the patient’s diagnosis.

**Table 1:** Patient's ED laboratory values.

Laboratory	Result	Reference value
White blood cell count	9000	4500–11,000/millmeters^3^
Hemoglobin	15.2	Male: 13.5–17.5 grams/deciliter
Aspartate aminotransferase (AST)	30	12–38 units/liter
Alanine aminotransferase (ALT)	30	10–40 units/liter
Potassium	4.0	3.5–5.0 milliequivalents/liter
Creatinine	1.09	0.6–1.2 milligrams/deciliter
Glucose	113	Random, non-fasting: <140 milligrams/deciliter
HIV screening	Negative	Negative

## Discussion

DCL is an uncommon clinical entity rarely found in the local population, as a result, there may be a delay in diagnosis without knowing the travel history and insect bite. A careful history can be used to identify possible etiologies of the cutaneous findings. In this case, the patient was in Honduras when he developed the ulcers. Since cutaneous leishmaniasis is endemic to this region, it should be suspected in individuals who are presenting with diffuse lesions and reported travel to this area. Identifying the etiology of skin ulcers was imperative in this case, as treatment of DCL is most often successful with one medication. The patient received ceftriaxone and TMP-SMX but had persistent and worsening symptoms prompting their visit to the ED. There was treatment failure, as DCL requires the antifungal, amphotericin B [5]. Studies have shown that treatments other than amphotericin B have been less effective [7]. Although the patient had a delay in diagnosis and treatment, several studies have shown that cutaneous leishmaniasis treated before the development of lesions may have a higher treatment failure rate [6,7]. Diagnosis of cutaneous leishmaniasis is made by skin biopsy, and the mainstay of treatment includes amphotericin B. This case highlights the importance of considering cutaneous leishmaniasis as a differential diagnosis in patients presenting with skin ulcers and a history of travel to endemic areas. The delay in diagnosis and treatment emphasizes the importance of additional education and research that is needed on the presentation of cutaneous leishmaniasis. Early diagnosis and appropriate treatment can improve patient outcomes and prevent complications.

## Conclusions

The patient had multiple evaluations by different providers before receiving the appropriate diagnosis and treatment for DCL. This case shows the difficulty in identifying DCL accurately. The patient required a multidisciplinary team and a biopsy for diagnosis before the patient was admitted for appropriate treatment. The gap between diagnosis and treatment highlights the need for additional education on DCL appearance even in unexpected locales.
